# Imbalance of Endocannabinoid/Lysophosphatidylinositol Receptors Marks the Severity of Alzheimer’s Disease in a Preclinical Model: A Therapeutic Opportunity

**DOI:** 10.3390/biology9110377

**Published:** 2020-11-05

**Authors:** Dina Medina-Vera, Cristina Rosell-Valle, Antonio J. López-Gambero, Juan A. Navarro, Emma N. Zambrana-Infantes, Patricia Rivera, Luis J. Santín, Juan Suarez, Fernando Rodríguez de Fonseca

**Affiliations:** 1 Instituto de Investigación Biomédica de Málaga-IBIMA, Unidad de Gestión Clínica de Salud Mental, Hospital Regional Universitario de Málaga, 29010 Málaga, Spain; cristina.rosell@ibima.eu (C.R.-V.); antonio.lopez@ibima.eu (A.J.L.-G.); juan_naga@hotmail.es (J.A.N.); patricia.rivera@ibima.eu (P.R.); juan.suarez@ibima.eu (J.S.); 2 Facultad de Ciencias, Universidad de Málaga, 29010 Málaga, Spain; 3 Facultad de Medicina, Universidad de Málaga, 29010 Málaga, Spain; 4 Departamento de Psicobiología y Metodología de las Ciencias del Comportamiento, Facultad de Psicología, Universidad de Málaga, 29010 Málaga, Spain; enzambrana@uma.es (E.N.Z.-I.); luis@uma.es (L.J.S.)

**Keywords:** Alzheimer’s disease, 5xFAD, endocannabinoid system, amyloid-β, neuroinflammation, GPR55

## Abstract

**Simple Summary:**

Alzheimer’s disease (AD) remains a major challenge for the healthcare system worldwide and, to date, no curative treatment is available. This disease is an irreversible progressive dementia that harms memory and cognitive functions, weakening the ability to carry out tasks by themselves. Among the potential targets for developing innovative therapies for AD, the endocannabinoid system has aroused much interest in the scientific community, since it is involved in multiple processes related to AD pathology. A major challenge to understand the role of the cannabinoid system in AD is to characterize how it contributes to the expression of a specific phenotype, from neuropathology to behavior. In the present study, we addressed this challenge by evaluating the expression of the endocannabinoid system in a transgenic mouse model of AD, bearing five familial AD mutations. Our data suggest that there is an association between the cannabinoid receptors and both the cognitive function and inflammatory response characterizing the disease. Moreover, this association is aggravated by genetic factors. From these data, the expression of endocannabinoid and G protein-coupled 55 receptors (GPR55), and endocannabinoid-related enzymes might be candidate markers for the detection of the severity of this neurodegenerative disease, eventually arising as potential therapeutic targets capable of modifying the course of this incapacitating dementia.

**Abstract:**

Alzheimer’s disease (AD) is the most common form of neurodegeneration and dementia. The endocannabinoid (ECB) system has been proposed as a novel therapeutic target to treat AD. The present study explores the expression of the ECB system, the ECB-related receptor GPR55, and cognitive functions (novel object recognition; NOR) in the 5xFAD (FAD: family Alzheimer’s disease) transgenic mouse model of AD. Experiments were performed on heterozygous (HTZ) and homozygous (HZ) 11 month old mice. Protein expression of ECB system components, neuroinflammation markers, and β-amyloid (Aβ) plaques were analyzed in the hippocampus. According to the NOR test, anxiety-like behavior and memory were altered in both HTZ and HZ 5xFAD mice. Furthermore, both animal groups displayed a reduction of cannabinoid (CB1) receptor expression in the hippocampus, which is related to memory dysfunction. This finding was associated with indirect markers of enhanced ECB production, resulting from the combination of impaired monoacylglycerol lipase (MAGL) degradation and increased diacylglycerol lipase (DAGL) levels, an effect observed in the HZ group. Regarding neuroinflammation, we observed increased levels of CB2 receptors in the HZ group that positively correlate with Aβ’s accumulation. Moreover, HZ 5xFAD mice also exhibited increased expression of the GPR55 receptor. These results highlight the importance of the ECB signaling for the AD pathogenesis development beyond Aβ deposition.

## 1. Introduction

Alzheimer’s disease (AD) is the most common form of progressive neurodegenerative disorder and dementia [1]. The neurodegeneration in AD is characterized by neuronal loss and synaptic failure resulting in cognitive impairment, memory loss, and behavioral changes [2]. AD has a clearly defined pathology signature, where dementia is associated with extracellular insoluble plaques composed of β-amyloid (Aβ) peptide, intracellular neurofibrillary tangles (NFTs) made of tau protein, and a glial inflammatory reaction that include astrogliosis and microglial cell proliferation [1,2]. Despite the research carried out, to date, no curative treatment is available, and it continues to be a challenge for scientists and clinicians searching for alternative therapeutic targets to prevent the development of cognitive impairment and dementia. Currently, the pharmacological treatments for patients with AD present two drawbacks: (1) they do not reverse disease progression and (2) they have several side effects, such as gastrointestinal, fatigue, dizziness, or headache [3,4]. Moreover, most AD cases are diagnosed too late, when the progression of the disease is already at an advanced stage [5]. Recent treatments for AD have been focused on lessening β-amyloid deposits. However, although relevant, amyloid deposition is only one part of a much larger collection of processes that lead to AD progression. As a clear example, extensive amyloid deposition in humans with a genetic inheritance of AD does not result in dementia if an additional mutation prevents a second process (tau deposition), which is also essential for neurodegeneration [6]. Therefore, an ideal treatment for AD should be able to modulate the disease through multiple mechanisms rather than targeting a single dysregulated pathway. 

Among the alternative targets explored in recent years for developing treatments against AD, the ECB system has risen much interest in the scientific community [7,8,9]. The ECB system is a bioactive lipid-based signaling system that consists of the cannabinoid receptors (CB1 and CB2) and proposed additional receptors such as the lysophosphatidylinositol (LPI) receptor GPR55, their endogenous ligands (anandamide (AEA) and 2-arachidonoylglycerol (2-AG)), and enzymes for its production (NAPE-PLD (*N*-acyl phosphatidylethanolamine-specific phospholipase D) and DAGL (diacylglycerol lipase)) and degradation (FAAH (fatty acid amidohydrolase) and MAGL (monoacylglycerol lipase)). Moreover, the ECB is involved in multiple processes in the central nervous system (CNS): synaptic plasticity, neuro-immunity, cognition, and emotional and neuroendocrine processes; it also reduces neuroinflammation, improves neurogenesis, and modulates amyloid neurotoxicity [10,11,12,13,14,15]. The cannabinoid receptor type 1 (CB1) is widely distributed in CNS neurons, especially in the cerebral cortex, hippocampus, basal ganglia, and cerebellum [16,17,18]. In contrast to the distribution of CB1 receptor, the cannabinoid receptor type 2 (CB2) is mainly located in immune system cells, whose brain representative is microglial cells. Upon microglial activation, CB2 receptors are densely expressed in microglia [19], acting as a feedback inhibitor of immune responsiveness in the CNS [10].

The ECB system has emerged as a novel therapeutic target to treat neurodegenerative diseases such as AD since it can modulate a range of aspects that occur in the disease [3,10]. The analysis of human postmortem samples of AD patients showed a decrease in the number of CB1-positive neurons in the frontal cortex in areas with amyloid plaques and activated microglial cells, as well as alterations of CB1 coupling to G proteins mediating its intracellular signaling [20,21]. However, these results are discussed since some studies have been reported ambiguous results on the expression and distribution of CB1 receptors in other areas such as the cortex and hippocampus in AD patients (for a review, see [8]). In addition, the role of interactions, in certain brain regions, between neurons expressing CB1 receptors and neurons expressing receptors of other systems may be relevant [22]. In any case, the study of the role of CB1 in AD is clearly supported by the crucial action of the CB1 receptor in cognitive processes such as learning and memory and the control of emotional behaviors such as anxiety, all of them crucial in the clinical evolution of AD patients [20,21,23]. Nevertheless, in AD patients, cognitive impairment has not been correlated with the expression of the CB1 receptors but with the presence and density of the plaques [21]. On the other hand, several reports have identified low expression of the CB2 receptor in some neurons [24,25,26]. Interestingly, the presence of microglial CB2 receptors surrounding Aβ plaques has been demonstrated [7,21]. Furthermore, there is a positive correlation among the quantity of CB2 receptors, Aβ42 peptide, and senile plaques in brain AD [21]. Moreover, recent findings have described the role of the CB2 receptor in the cognitive functions in different transgenic mouse models of AD [27,28,29,30]. However, in AD patients, no correlation between CB2 receptor levels and the cognitive status was found [21]. 

In addition to CB1 and CB2 cannabinoid receptors, there are other G protein-coupled receptors capable of being activated by endogenous and synthetic cannabinoids. One of these emerging receptors is the orphan receptor 55 (GPR55), a proposed LPI receptor. It is highly expressed in the CNS and can be also activated by cannabinoids, emerging as a putative “type 3” cannabinoid receptor [31]. GPR55 receptor plays a crucial role in anxiety [32] and behaviors related to hippocampal activity [33].

Despite their role in neuronal communication, the high presence of cannabinoid receptors in glial cells support their role as an anti-inflammatory and neuroprotective mechanism in the CNS [34,35,36]. AEA and 2-AG are released in response to pathogenic events, i.e., the ECB system is activated on demand, providing compensatory responses linked to neuronal repair and cell maintenance [15]. In this sense, under inflammatory conditions, both microglia and astrocytes synthesize these endocannabinoids in the brain via the catalytic action of DAGL and NAPE-PLD [13]. Recent data have demonstrated the link between MAGL, the main enzyme responsible for the degradation of 2-AG, and neuroinflammation in a mouse model of AD [9,37]. Moreover, recent data have corroborated that deletion of FAAH, which hydrolyzes AEA, led to a proinflammatory phenotype [38] and significant changes in the amyloid pathophysiology of 5xFAD mice [36]. Therefore, growing data suggest that ECB enzymes may be also implicated in the aggregation and degradation of the toxic peptide and could modulate binding and signaling through ECB receptors, a fact that could be relevant for the interaction between the ECB system and AD.

Consequently, the use of transgenic animal models of AD, for instance, the transgenic 5xFAD animal model [39] could bring knowledge about the contribution of the endocannabinoid system components to cognitive abilities and physiological role in the hippocampus. The 5xFAD mice (Tg6799 line) represent one of the most aggressive and early-onset amyloid pathology models, exhibiting neuroinflammation (astrogliosis and microgliosis) in response to amyloid deposition [39,40,41] unlike other transgenic animals models (for a review, see [42]). In addition, 5xFAD mice had significant neuronal loss by 9–12 months of age [39,43,44] and cognitive decline starting at 4 months [39,45], which increased substantially with age [46,47]. Regarding the endocannabinoid system, Lopez et al. (2018) demonstrated the increase in expression of CB2 receptor in areas with intense inflammation and amyloid deposits such as the cortex, hippocampus, brain stem, and thalamus in the 5xFAD model with enhanced green fluorescent protein-tagged CB2 receptor [7], corroborating the results found in AD patients [21]. However, to date, no wide analysis of the expression of ECB-related proteins, including GPR55 receptor expression, has been addressed in this model. In the present study, we explore all the ECB system components in the hippocampus of the transgenic 5xFAD mouse model, taking into consideration the genetic load of the disease: heterozygous (HTZ) versus homozygous (HZ) conditions. Initially, we evaluated the anxiety-like behavior and memory at 11 months old using the NOR test. Second, we mainly analyzed the ECB system components and neuroinflammatory markers, as well as the β-amyloid accumulated in the hippocampus. The outcome of this study aims to highlight the association of the ECB with the 5xFAD phenotype as a basis for establishing the potential utility of the ECB system as a novel therapeutic target to treat AD.

## 2. Materials and Methods

### 2.1. Animals and Ethics Statement

We used 5xFAD (FAD: familial Alzheimer’s disease) APP/PS1 double transgenic mice that co-express and co-inherit FAD mutant forms of human APP (the Swedish mutation: K670N, M671L; the Florida mutation: I716V; the London mutation: V717I) and PS1 (M146L; L286V) transgenes under transcriptional control of the neuron-specific mouse Thy-1 promoter (Tg6799 line) [39,40,41]. These mice co-express mutations of the human genes encoding the amyloid protein precursor (APP) and presinilin1 (PS1), increasing the production of 42-amino-acid β-amyloid (Aβ42). All experiments were done with females and males at 11 months of age and were realized in compliance with the ARRIVE guidelines [48] and in concordance with the European Communities Council Directives 2010/63/EU, Regulation (EC) No. 86/609/ECC (24 November 1986) and Spanish National and Regional Guidelines for Animal Experimentation (Real Decreto 53/2013). 5xFAD mice used were heterozygous (HTZ) (*n* = 7; four males and three females) and homozygous (HZ) (*n* = 12; seven males and five females) concerning the transgene, and nontransgenic wild-type (No-Tg) (*n* = 13; five males and eight females) littermate mice served as controls. We did not perform a sex analysis because of the reduced number of samples per genotype and because our main goal was to evaluate the differences according to heterozygous and homozygous conditions. Experimental protocols were approved by the Local Ethical Committee for Animal Research of the University of Malaga (CTS-8221, July 2016). Accordingly, all efforts were made to minimize animal suffering and to reduce the number of animals used.

### 2.2. Novel Object Recognition Test (NOR)

**Figure 1:**
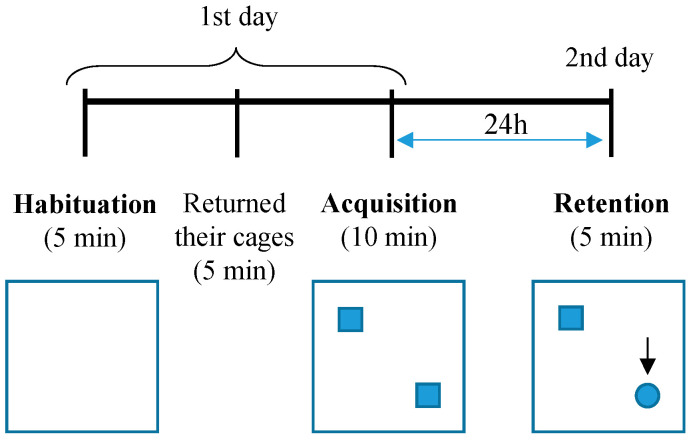
Schematic representation of novel object recognition assay. Following habituation trial in the empty boxes for 5 min, mice were allowed to explore an identical pair of objects placed in the boxes for 10 min as the acquisition trial. After a 24 h stay in the home cage, the mice were returned to the arena where two objects, one familiar and one novel, were placed. The time that mice spent exploring the two objects was recorded. The arrow indicates the novel object in the box. Squares symbolize the object number one (familiar object); the circle symbolizes the object number two (novel object).

The NOR test was used to analyze long-term memory. The test (Figure 1) consisted of four open-field apparatus (40 × 40 cm, made of gray Plexiglas), and mice were placed in the center of the arena to explore freely for 5 min (habituation trial). Then, animals were returned to their cages for 5 min. In the acquisition trial, two identical objects were presented on the up and down corners (the left–right position was counterbalanced) of the apparatus, and mice explored both objects for 10 min. Twenty-four hours later, mice were returned to the arena and were presented with one familiar object and a novel one that allowed them to explore for 5 min [49]. We analyzed locomotion (distance moved; cm) and anxiety-like behavior (time spent in the center of the apparatus). During acquisition and retention trials, the total time spent exploring objects (i.e., touching them with the nose or forepaws, analyzed observationally) was recorded. To assess cognitive performance, the percentage of novelty preference was calculated as follows: (the time spent exploring novel object / the time spent exploring both objects) × 100 [50].

### 2.3. Tissue Processing and Western Blot Analysis

#### 2.3.1. Brain Protein Extract

All mice were sacrificed at 11 months of age. The brain was removed and bisected down the midline; one hemibrain was used for histological procedures and stored in 4% paraformaldehyde (PFA) and the other hemibrain was kept in dry ice for storage at −80 °C for biochemical analysis. Hippocampus samples (17 mg per sample) were dissected and homogenized in 1 mL of cold RIPA lysis buffer (50 mM Tris-HCl pH 7.4, 150 mM NaCl, 0.5% NaDOC, 1 mM EDTA, 1% Triton, 0.1% SDS, 1 mM Na_3_VO_4_, 1 mM NaF) supplemented with a protease cocktail (Hoffmann-La Roche, Basel, Switzerland). The suspension was incubated for 2 h at 4 °C, followed by centrifugation at 12,000 rpm for 15 min at 4 °C. The supernatant was transferred to a new clean centrifuge tube, and the Bradford colorimetric method was used to determine the concentration of the total protein. The protein extracts were diluted 1:1 in loading buffer (DTT 2X) and heated for 5 min at 99 °C before being subjected to electrophoresis.

#### 2.3.2. Western Blot Analysis

The protein expression of ECB system components was analyzed using the Western blot technique. To this end, CB1 (1:100, Abcam, Cambridge, United Kingdom), CB2 (1:200, Abcam), and GPR55 (1:500, Abcam) receptors were measured. Furthermore, NAPE-PLD (1:1000, Abcam) and DAGLα/β (1:100, bioNova, Helsinki, Finland) were quantified as production enzymes, and FAAH (1:100, Cayman Chemical, Ann Arbor, MI, USA) and MAGL (1:500, Cayman Chemical, Ann Arbor, MI, USA) were quantified as degradation enzymes. Neuroinflammation was also studied through the expression of iNOS (1:200, Thermo Fisher Scientific, Waltham, MA, USA), COX-2 (1:200, Cell Signaling, Danvers, MA, USA), Iba1 (1:500, Wako, Richmond, VA, USA), and GFAP (1:500, Sigma-Aldrich, San Luis, MO, USA) (see Table S1, for additional manufacturing information). Experimental groups (No-Tg, HTZ, HZ) consisted of six animals. The tissue protein (10–15 μg) was subjected to electrophoresis on 4–12% Criterion XT Precast Bis-Tris gels (Bio-Rad, Hercules, CA, USA) for 30 min at 80 V and 2 h at 150 V. Proteins were transferred onto a 0.2 µm nitrocellulose membrane (Bio-Rad, USA) for 1 h at 80 V using wet transfer equipment. The membrane was washed twice for 5 min in TBS-T (10 mM Tris–HCl, 150 mM NaCl, 0.1% Tween 20, pH 7.6) and blocked with 2% bovine serum albumin/Tris-buffered saline + Tween 20 (BSA–TBST) for 1 h at room temperature on a shaker platform. Subsequently, the membrane was incubated with respective primary antibodies overnight at 4 °C diluted in 2% BSA–TBST. The following day, the membrane was washed three times for 5 min with TBST. An appropriate HRP-conjugated rabbit/mouse secondary antibody (Promega, Madison, WI, USA) was diluted 1:10,000 in 2% BSA–TBST and incubated with the membrane for 1 h shaking at room temperature. Finally, the membrane was washed as above and exposed to a chemiluminescent reagent (Santa Cruz, Biotechnology Inc., Dallas, TX, USA) for 5 min. Stripping/reproving steps were used when necessary. Respective membrane-bound protein was then visualized by chemiluminescence (ChemiDoc Imaging System, Bio-Rad, Hercules, CA, USA). Bands were quantified by densitometric analysis using ImageJ software (Rasband, WS, ImageJ, US National Institutes of Health, Bethesda, MD, USA). Normalization was performed using a reference protein of the same membrane, α-adaptin. The results were expressed as the protein/α-adaptin ratio or phosphorylated/total protein ratio and normalized to the control group (*y*-axis represents “fold mean of the control values”).

### 2.4. Immunohistochemical Analysis

The hemibrains were fixed in 0.1 M PBS containing 4% PFA for 48 h and cryopreserved in 30% sucrose in 0.1 M PBS solution for 5 days at 4 °C. Hemibrains were cut at a 50 µm thickness in the coronal plane on a microtome (Microm HM460, Microm Laborgerate S.I., Barcelona, Spain), and sections from heterozygous and homozygous 5xFAD mice and non-transgenic mice were performed as previously described in [51]. Serial sections were blocked with 5% donkey serum and 0.5% Triton X-100 in 0.1 M PBS for 45 min at room temperature. For plaque amyloid-β analysis, we used rabbit anti-Aβ (1:500, Abcam), rabbit anti-amyloid β1–40 (1:200, ThermoFisher), and rabbit anti-Aβ1–42 (1:500, ThermoFisher). For neuroinflammation analysis, we used rabbit anti-glial fibrillary acidic protein (GFAP; 1:1000, Dako Agilent, Santa Clara, CA, USA) and rabbit anti-Iba1 (1:500, Abcam). Primary antibodies were incubated overnight at room temperature. After rinsing, the sections were incubated with secondary antibody biotinylated goat anti-rabbit (1:500, GE Healthcare, Chicago, IL, USA) for 2 h at room temperature. All antibodies were diluted in PBS, 0.5% Triton X-100, and 2.5% donkey serum (Sigma- Aldrich). We used the peroxidase-conjugated ExtrAvidin method and diaminobenzidine as the chromogen to visualize the reaction product.

### 2.5. Plaque and Microglial Cell Activation and Neuron Quantification in the Hippocampus

The number of labeled Aβ plaques, as well as GFAP- and Iba1-positive cells, was quantified in the hippocampus. Images were acquired with digital Camera DP70 (Olympus Iberia, S.A., Barcelona, Spain) connected to a microscope Olympus BX41. For immunostaining quantification, ImageJ software was used [52]. The images were binarized to 16 bit black, and a fixed intensity threshold was applied for each immunostaining. Eight mice per group and three sections per mouse at three different hippocampal levels were used.

### 2.6. Statistical Analyses

Statistical analysis was conducted in GraphPad Prism, version 8 (GraphPad Software, Inc., La Jolla, CA, USA). The Shapiro–Wilk test was used to assess normal distribution of data. Levene’s test was used to analyze the assumption of homogeneity of variance. One-way and two-way (“genetic × object”) analysis of variance (ANOVA) was assessed for behavioral tests, Western blot, and immunostaining quantification followed by a Tukey’s multiple-comparisons test. The post hoc tests were conducted only if F in ANOVA achieved a *p*-value less than 0.05 and there was no statistically significant variance inhomogeneity. The analysis of two single groups was performed using Student’s unpaired *t*-test. Correlation analysis was performed computing the value of the Pearson correlation coefficient, *r* (its value ranges from −1 to +1). All data were expressed as the mean ± standard error of the mean (SEM), and *p*-values less than 0.05 were considered significant.

## 3. Results

### 3.1. Both Heterozygous and Homozygous 5xFAD Transgenic Mice Have Impairment in Novel Object Recognition

**Figure 2:**
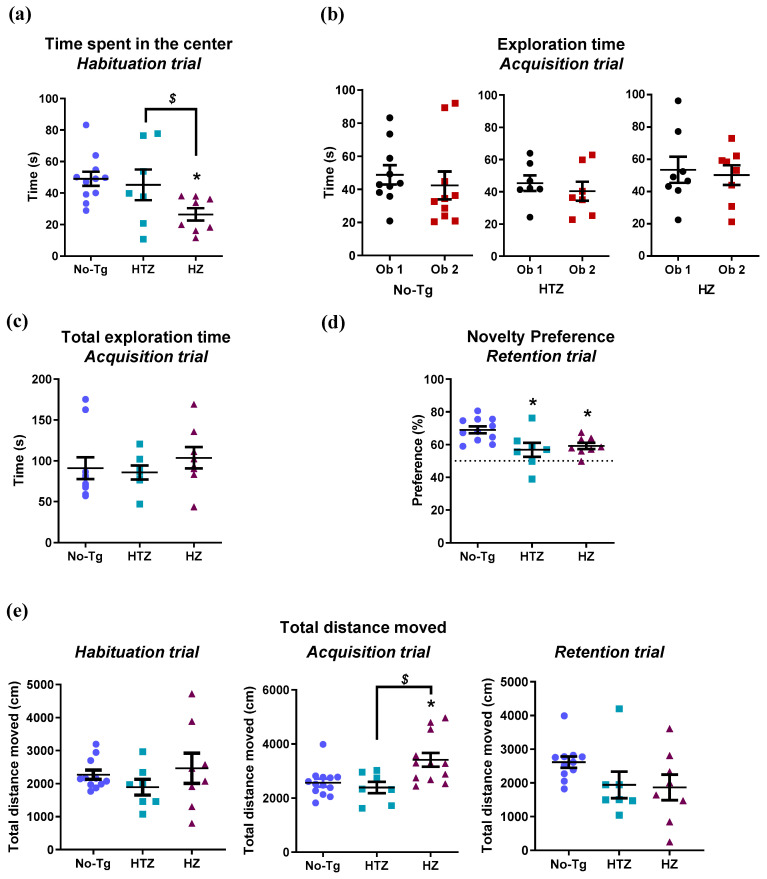
Impaired memory in both heterozygous (HTZ) and homozygous (HZ) 5xFAD (FAD: family Alzheimer’s disease) transgenic mice at 11 months of age on the novel recognition memory. (**a**) HZ 5xFAD mice spent less time in the center throughout the habituation trial. No differences were found (**b**) in exploring both objects or (**c**) in the total exploration time. (**d**) Both HTZ and HZ 5xFAD mice exhibited impaired memory in the retention trial. (**e**) There were no differences in locomotion between genotypes in the habituation and retention trial, but HZ 5xFAD mice exhibited hyperactivity in the acquisition trial compared to No-Tg and HTZ. One-way ANOVA and Tukey’s test were performed: (*) *p* < 0.05 vs. nontransgenic wild-type (No-Tg) group; ($) *p* < 0.05 between HTZ and 5xFAD group. All data are displayed as the mean ± standard error of the mean (SEM).

First, we evaluated long-term memory using the novel object recognition task. In the habituation trial, homozygous mice showed less time spent in the center of the arena compared to their heterozygous and nontransgenic littermates (one-way ANOVA: F_(2,22)_ = 4.110, *p =* 0.0298; Tukey’s test: *p* ≤ 0.05; Figure 2a), suggesting an anxiety-like behavior. In the acquisition trial, all animals explored both objects without presenting a marker preference for any ones (two-way ANOVA: “genotype” effect: F_(2,44)_ = 0.8665, *p =* 0.4275; “object” effect: F_(1,44)_ = 0.7382, *p =* 0.3949, Figure 2b), and there was no significant difference between genotypes in the total exploration time (one-way ANOVA: F_(2,22)_ = 0.5010, *p =* 0.6127; Figure 2c). However, in the retention trial, where one of the objects was novel, both heterozygotes and homozygotes 5xFAD mice exhibited a lower percentage of novelty preference compared to control mice (one-way ANOVA: F_(2,23)_ = 5.940, *p =* 0.0083; Tukey’s test: *p* ≤ 0.05; Figure 2d). No significant differences were observed in locomotion (distance moved) during the habituation or retention trial between genotypes (one-way ANOVA: habituation trial: F_(2,22)_ = 1.036, *p =* 0.3717; retention trial: F_(2,22)_ = 2.174, *p =* 0.1375; Figure 2e). However, in the acquisition trial, locomotion observed in the HZ 5xFAD mice was significantly higher compared to the No-Tg and HTZ group (one-way ANOVA: F_(2,23)_ = 5.288, *p =* 0.0129; Figure 2e).

### 3.2. Hippocampal Alteration of the Expression of CB1, CB2, and GPR55 Receptors in Homozygous 5xFAD Transgenic Mice

**Figure 3:**
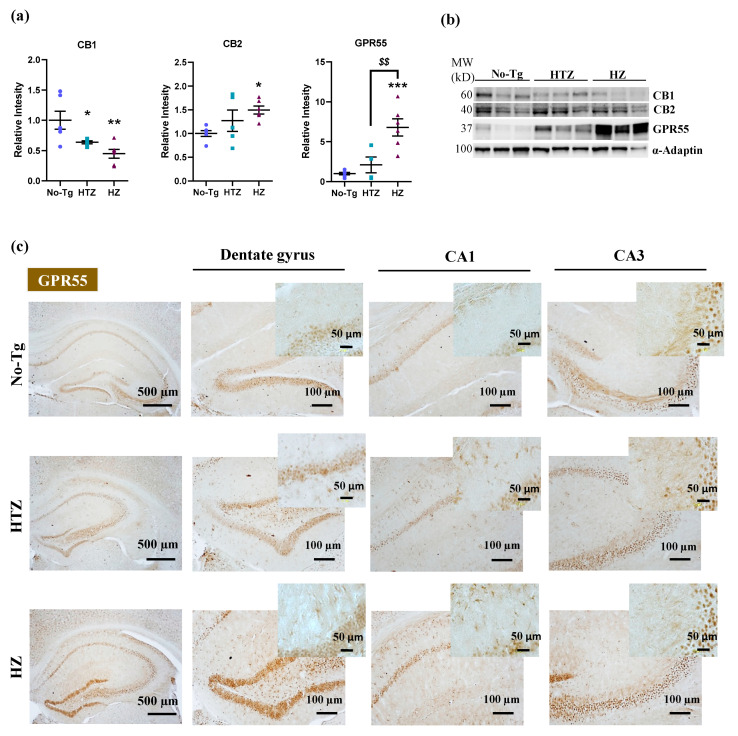
Effect of the genetic load of the disease on protein expression of the endocannabinoid system in a transgenic mouse model of Alzheimer’s disease (AD) (5xFAD). (**a**) Bar charts represent the quantification of Western blot membranes (**b**) after incubating with anti-cannabinoid receptor (CB1, CB2) and anti-G-protein-coupled receptor 55 (GPR55) antibodies and normalized with α-adaptin. We performed the Western blot analysis on hippocampus homogenates. The blots shown in (**b**) are a representation of all the bands (see Figures S2 and S3, for additional information), resulting from three out of six independent samples (three out of five for the HTZ group). Molecular weights (MW) are indicated in kilodaltons (kDa). The corresponding expression of α-adaptin is shown as a loading control per lane. (**c**) The images show the representative GPR55 staining in the hippocampus, dentate gyrus, CA1, and CA3 of each group. One-way ANOVA and Tukey’s test were performed: (*) *p* < 0.05, (**) *p* < 0.01 and (***) *p* < 0.001 vs. No-Tg group; ($$) *p* < 0.01 between HTZ and 5xFAD group. Histograms represent the mean ± SEM (*n* = 6) Scare bar = 500, 100 and 50 µm.

We next analyzed the expression of the cannabinoid receptors (CB1, CB2, and GPR55 receptors) by Western blotting in the hippocampus of 5xFAD transgenic mice, as it is a key structure for learning and memory function. Figure 3a shows the quantification of Western blot membranes (Figure 3b) after incubating with the selected antibodies labeling those lipid receptors. Intriguingly, HTZ and HZ 5xFAD animals exhibited a clear reduction in the expression of CB1 receptors (one-way ANOVA: F_(2,14)_ = 7.948, *p* = 0.0049; Tukey’s test: *p* ≤ 0.05; Figure 3a,b) compared to the No-Tg mice, suggesting that this decrease may be related to memory dysfunction. Regarding the expression of CB2 receptors, there was a remarkable increase in the expression in homozygous 5xFAD mice compared to the No-Tg group (one-way ANOVA: F_(2,14)_ = 3.868, *p =* 0.0460; Tukey’s test: *p* ≤ 0.05; Figure 3a,b).

In addition to CB1 and CB2 receptors, the GPR55 receptor is proposed as a cannabinoid receptor involved in the modulation of neuroinflammation [31]. As shown in the Western blot analysis, GPR55 is highly expressed in homozygous 5xFAD mice (one-way ANOVA: F_(2,13)_ = 15.15, *p =* 0.0004; Tukey’s test: *p* ≤ 0.01; Figure 3a,b) compared to HTZ and No-Tg mice. We also performed an immunohistochemistry assay to analyze the expression of GPR55 in the hippocampus, especially in the HZ 5xFAD mice (Figure 3c). Qualitatively, the higher presence of GPR55 was observed in HZ 5xFAD mice in both dentate gyri, CA1 and CA3, compared to the control group. These results validated the data found by Western blot (see Figure S1, for GPR55 immunofluorescence staining).

To establish a relationship between the main changes found in endocannabinoid receptors and the memory-related behavioral test, we performed correlation studies (Table 1). First, the time spent in the center of the open field test in the habituation trial negatively correlated with the quantity of CB2 and GPR55 receptors (CB2: Pearson’s *r =* −0.523, *p* < 0.0001; GPR55: Pearson’s *r =* −0.750, *p* < 0.0001). No correlation was found with CB1 (Pearson’s *r =* 0.589, *p =* 0.72550). Next, we evaluated locomotion as distance moved in the acquisition trial, and we found that negatively correlated with CB1 (Pearson’s *r =* 0.479, *p =* 0.00210) and positively correlated with GPR55 (Pearson’s *r =* 0.680, *p =* 00249). No correlation was found with CB2 (Pearson’s *r =* 0.445, *p =* 0.45070). Lastly, the percentage of novelty preference during the retention trial positively correlated with CB1 (Pearson’s *r =* 0.634, *p =* 0.03950), together with a negative correlation with the quantity of CB2 and GPR55 receptors (CB2: Pearson’s *r =* −0.483, *p =* 0.0034; GPR55: Pearson’s *r =* −0.525, *p =* 0.0126).

### 3.3. Alteration in the Endocannabinoid Production and Degradation Pathways in Homozygous 5xFAD Transgenic Mice

**Figure 4:**
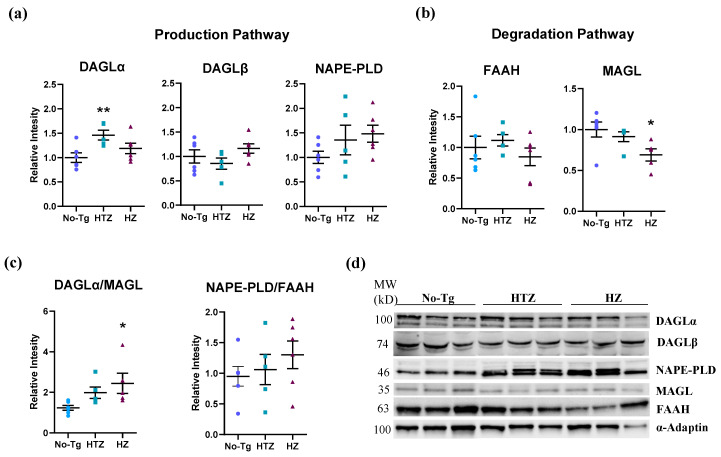
Production and degradation pathways in 5xFAD transgenic mice. Bar charts in (**a**,**b**) represent the quantification of western blot membranes (**d**) after incubating with anti-diacylglycerol lipase alpha (DAGLα), DAGLβ, *N*-acyl phosphatidylethanolamine-specific phospholipase D (NAPE-PLD), fatty acid amide hydrolase (FAAH), and monoacylglycerol lipase (MAGL) antibodies and normalized with α-adaptin. Bar charts in (**c**) are the ratio between DAGLα and MAGL and between NAPE-PLD and FAAH, both previously normalized with α-adaptin. We performed the Western blot analysis on hippocampus homogenates. The blots shown in (**d**) are a representation of all the bands (see Figures S2 and S4, for additional information), resulting from three out of six independent samples (three out of five for the HTZ group). Molecular weights (MW) are indicated in kilodaltons (kDa). One-way ANOVA and Tukey’s test were performed: (*) *p* < 0.05 and (**) *p* < 0.01 vs. No-Tg group. Histograms represent the mean ± SEM (*n* = 6).

We performed a Western blot analysis to study the expression of the enzymes DAGL and NAPE-PLD involved in the biosynthesis of the endocannabinoids 2-AG and AEA. Heterozygous 5xFAD transgenic mice increased the expression of DAGLα (one-way ANOVA: F_(2,14)_ = 4.811, *p =* 0.0257; Tukey’s test: *p* ≤ 0.01; Figure 4a,d) compared to the No-Tg group. Despite showing a certain rise in protein quantity, DAGLβ (one-way ANOVA: F_(2,14)_ = 1.672, *p =* 0.2232; Figure 4a,d) and NAPE-PLD (one-way ANOVA: F_(2,14)_ = 1.618, *p =* 0.2333; Figure 4a,d) did not show a significant statistical increase.

Regarding the degradation enzymes, we did not observe differences between genotype in the expression of fatty acid amide hydrolase (FAAH) enzyme (one-way ANOVA: F_(2,14)_ = 0.7668, *p =* 0.4830; Figure 4b,d). However, the expression of monoacylglycerol lipase (MAGL) enzyme was decreased in the homozygous 5xFAD group (one-way ANOVA: F_(2,13)_ = 4.059, *p =* 0.0427; Tukey’s test: *p* ≤ 0.05; Figure 4b,d) compared to the control group. 

To study whether there were more production or degradation enzymes, we performed a ratio between both enzymes. First, we compared the 2AG production and degradation enzymes (DAGLα versus MAGL) and it showed an increase in DAGLα in homozygous 5xFAD transgenic mice (one-way ANOVA: F_(2,13)_ = 3.818, *p =* 0.0496; Tukey’s test: *p* ≤ 0.05; Figure 4c). On the other hand, the ratio of AEA production and degradation enzymes (NAPE-PLD versus FAAH) did not show a significant difference among groups (one-way ANOVA: F_(2,14)_ = 0.7735, *p =* 0.4802; Figure 4c).

### 3.4. Neuroinflammatory Response in Both Heterozygous and Homozygous 5xFAD Transgenic Mice—Stronger in the Homozygous Group

**Figure 5:**
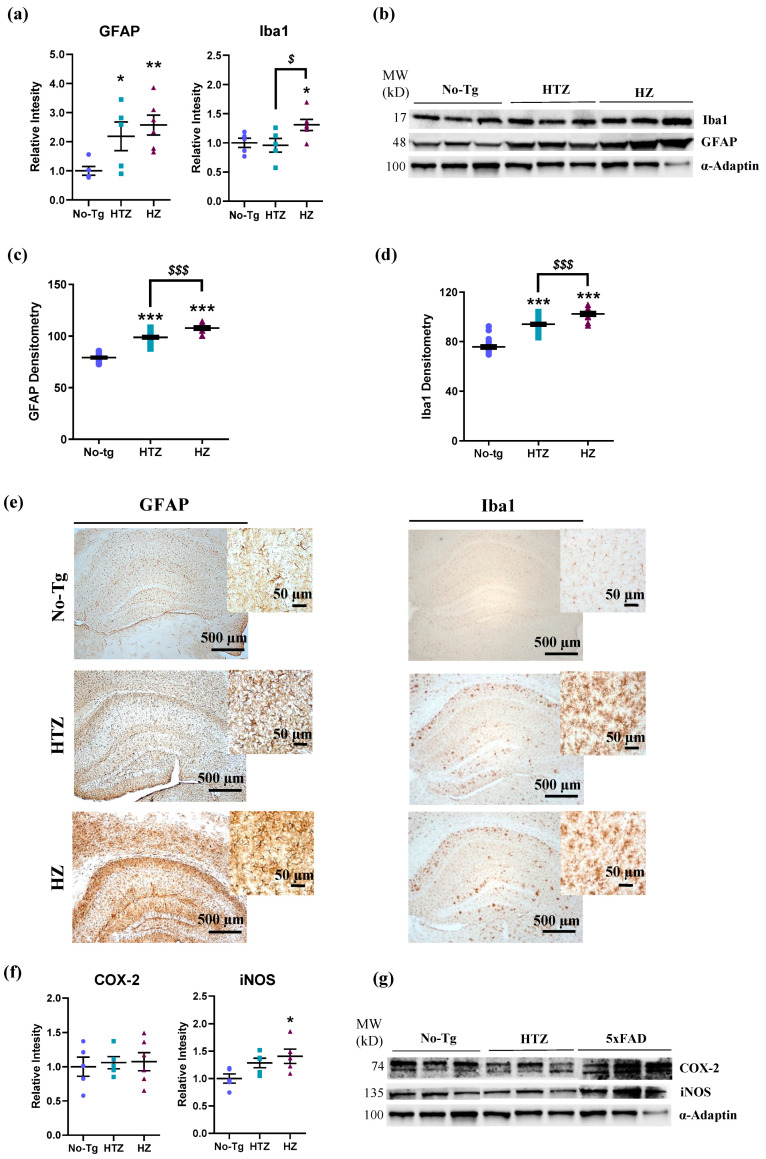
Neuroinflammation response in both heterozygous and homozygous 5xFAD transgenic mice. Bar charts in (**a**) represent the quantification of Western blot membranes (**b**) after incubating with anti-glial fibrillary acidic protein (GFAP) and ionized calcium-binding adaptor molecule 1 (Iba1), and normalized with α-adaptin. Bar charts in (**c**,**d**) represent the quantification of GFAP and Iba1 immunostaining (**e**) in the hippocampus of each group. Bar charts in (f) represent the quantification of Western blot membranes (**g**) after incubating with cyclooxygenase 2 (COX-2) and inducible nitric oxide synthase (iNOS) antibodies, and normalized with α-adaptin. We performed the Western blot analysis on hippocampus homogenates. The blots shown in (**b**,**g**) are a representation of all the bands (see Figures S3–S5, for additional information), resulting from three out of six independent samples (three out of five for the HTZ group). Molecular weights (MW) are indicated in kilodaltons (kDa). One-way ANOVA and Tukey’s test were performed: (*) *p* < 0.05, (**) *p* < 0.01 and (***) *p* < 0.001 vs. No-Tg group; ($) *p* < 0.05 and ($$$) *p* < 0.001 between HTZ and 5xFAD group. Histograms represent the mean ± SEM (*n* = 6). Scale bar = 500 and 50 µm.

In the next set of experiments, we used immunohistochemistry to evaluate the neuroinflammatory response. The analysis of GFAP and Iba1 protein expressions in the hippocampus by Western blot showed that both were significantly increased in HTZ and in HZ 5xFAD mice compared to the No-Tg group (one-way ANOVA: F_(2,13)_ = 5.275, *p =* 0.0210; Tukey’s test: *p* ≤ 0.05; GFAP; one-way ANOVA: F_(2,13)_ = 3.985, *p =* 0.0447; Tukey’s test: *p* ≤ 0.05; Iba1; Figure 5a,b). These expressions were validated by immunochemistry (Figure 5c). GFAP and Iba1 densitometry confirmed the higher expression in HTZ and HZ 5xFAD transgenic mice compared to control mice (GFAP: one-way ANOVA: F_(2,39)_ = 129.8, *p* < 0.0001; Tukey’s test: *p* ≤ 0.001; Iba1: one-way ANOVA: F_(2,61)_ = 88.24, *p* < 0.0001; Tukey’s test: *p* ≤ 0.001; Figure 5c,d).

On the other hand, inflammatory mediators such as inducible nitric oxide synthase (iNOS) and cyclooxygenase (COX-2) were also evaluated by Western blot analysis. We did not observe a change in COX-2 protein expression between groups (one-way ANOVA: F_(2,13)_ = 0.09904, *p =* 0.9064; Figure 5f,g). However, iNOS protein expression was significantly increased in homozygous 5xFAD transgenic mice (one-way ANOVA: F_(2,12)_ = 4.126, *p =* 0.0433; Tukey’s test: *p* ≤ 0.05; Figure 5f,g) compared to No-Tg mice.

The potential relationship of the ECB receptors (CB1, CB2, and GPR55) and neuroinflammation response exhibited by animals was explored (Table 2). The expression of CB1 receptors negatively correlated with all the four inflammatory markers analyzed (Iba1: Pearson’s *r* = −0.513, *p =* 0.003; GFAP: Pearson’s *r =* −0.722, *p* < 0.0001; COX2: Pearson’s *r* = −0.717, *p* = 0.0006; iNOS: Pearson’s *r =* −0.724, *p* < 0.0001). On the other hand, the expression of CB2 receptors correlated positively with GFAP and COX2 (GFAP: Pearson’s *r* = 0.583, *p =* 0.0062; COX2: Pearson’s *r =* 0.576, *p =* 0.0446), while no correlation was found with Iba1 and iNOS (Iba1: Pearson’s *r =* 0.470, *p =* 0.9685; iNOS: Pearson’s *r =* 0.583, *p =* 0.716). Lastly, GPR55 negatively correlated with Iba1, COX2, and iNOS (Iba1: Pearson’s *r =* −0.704, *p =* 0.0089; COX2: Pearson’s *r =* −0.696, *p =* 0.0029; iNOS: Pearson’s *r =* −0.729, *p =* 0.0065), and no correlation was observed with GFPA (Pearson’s *r =* −0.656, *p =* 0.1453).

### 3.5. Neuroinflammatory Response in Both Heterozygous and Homozygous 5xFAD Transgenic Mice—Stronger in the Homozygous Group

**Figure 6:**
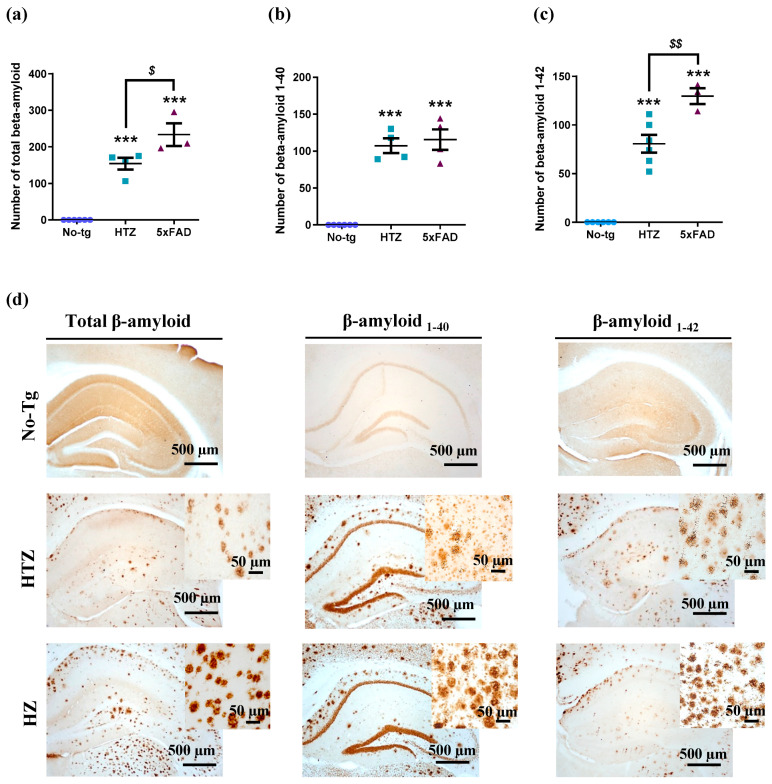
Amyloid-β (Aβ) accumulation in the hippocampus of heterozygous and homozygous 5xFAD transgenic mice. Quantification of (**a**) total β-amyloid, (**b**) β-amyloid 1–40, and (**c**) β-amyloid 1–42 in the hippocampus of three groups. HZ 5xFAD mice exhibited a higher accumulation of total β-amyloid and β-amyloid 1–42 compared to HTZ mice. (**d**) The images correspond to representative total β-amyloid, β-amyloid 1–40 and β-amyloid 1–42 staining of the hippocampus of each group. Histograms represent the mean ± SEM (*n* = 6). One-way ANOVA and Tukey’s test were performed: (***) *p* < 0.001 vs. No-Tg group; ($) *p* < 0.05 and ($$) *p* < 0.01 between HTZ and 5xFAD group. Scale bar = 500 and 50 µm.

To evaluate the β-amyloid burden, the number of labeled Aβ plaques was quantified in the hippocampus of 5xFAD transgenic mice. Both HTZ and HZ 5xFAD transgenic mice exhibited a similar accumulation of β-amyloid in the hippocampus (Figure 6), and it was significantly higher than No-Tg mice which had an absence of plaques (one-way ANOVA: F_(2,10)_ = 70.03, *p* < 0.0001; Tukey’s test: *p* ≤ 0.001; Figure 6). In the same sense, we did not observe differences between heterozygous and homozygous 5xFAD transgenic mice in Aβ1–40 although it was significantly different from the control group (one-way ANOVA: F_(2,11)_ = 66.57, *p* < 0.0001; Tukey’s test: *p* ≤ 0.001; Figure 6). Interestingly, there was a higher accumulation of Aβ1–42 peptide in homozygous 5xFAD mice than heterozygous mice (one-way ANOVA: F_(2,12)_ = 80.68, *p* < 0.0001; Tukey’s test: *p* ≤ 0.001; Figure 6) suggesting a more severe neuropathological phenotype.

On another note, the correlation studies (Table 3) revealed that the number of Aβ plaques was negatively associated with the expression of CB1 receptors (Aβ1–40: Pearson’s *r* = −0.700, *p <* 0.0001; Aβ1–42: *r* = −0.723, *p <* 0.0001; Aβtotal: *r* = −0.717, *p <* 0.0001) and positively with the expression of CB2 receptors (Aβ1–40: Pearson’s *r* = 0.553, *p <* 0.0001; Aβ1–42: *r* = 0.587, *p <* 0.0001; Aβtotal: *r* = 0.575, *p <* 0.0001). No correlation was observed between GPR55 receptor and amyloid-β burden (Aβ1–40: Pearson’s *r* = 0.626, *p* = 0.4077; Aβ1–42: *r* = 0.712, *p* = 0.4806; Aβtotal: *r* = 0.681, *p* = 0.4652).

## 4. Discussion

The present study allowed us to holistically analyze the expression of the ECB system in the hippocampus of a genetic model of human AD, under conditions of hetero- and homozygosis. AD pathophysiology is well known for the accumulation of senile plaques and reactive gliosis, among other events that develop especially in the hippocampus [1,2]. The present data firmly support the recent publications on the association of the ECB system and AD [53], which adds a set of new biomarkers related to the severity of the phenotype to the classical neuropathological analysis. In this sense, the major findings of our study are the differences observed in the expression pattern of CB1, CB2, and GPR55 receptors, as well as the MAGL degrading enzyme, according to the transgenic load in the 5xFAD transgenic mouse model at 11 months of age. Here, our data show ongoing ECB system alterations in the hippocampus reflected by an elevated neuroinflammatory response induced by β-amyloid burden consistent with memory impairment aggravated by homozygosity. To our knowledge, we are the first to report an association between cannabinoid molecules and the neuropathological hallmarks of AD studied in the 5xFAD line, taking special consideration of the transgenic load: heterozygous versus homozygous condition. This is a relevant finding since the heterozygosity condition might allow space for analyzing disease-modifying factors (i.e., the impact of accelerating factors that promote more severe disease progression).

In the 5xFAD mice model (homozygous condition), it has been demonstrated that memory deficit occurs from 5–6 months of age [39] and increases substantially with age [46,47], caused by the accumulation of β-amyloid plaques. However, the biological mechanisms involved in the detriment of pathology are still unclear. Our data show that both HTZ and HZ 5xFAD mice exhibited a significant decrease in the percentage of novelty in NOR indicating hippocampus-dependent memory impairment at 11 months of age. Moreover, there are only a few studies that focused on heterozygosity as a useful tool to understand AD. In this context, Richard et al. (2015) informed that 5xFAD mice, when bred to homozygosity, presented a significant age-dependent motor phenotype and spatial reference memory assessed by Morris water maze compared to the heterozygous condition at 5 months of age [54]. Otherwise, our data revealed that HTZ 5xFAD mice exhibited an impaired memory in NOR at 11 months of age, and it could be associated with the enhanced number of Aβ deposits throughout the hippocampus, as shown by our results analyzed using immunohistochemistry. In this sense, it is important to note that the transgenic load affects the ECB system in a different way, exacerbating emotional and memory function in this transgenic mouse model of AD. Therefore, the 5xFAD mouse model (heterozygous and homozygous condition) could elucidate the role(s) of cannabinoid and noncannabinoid receptors in the development of AD. Potential experimental analysis of new cannabinoid receptor ligands, FAAH inhibitors, or even DAGL inhibitors might benefit from this approach with heterozygous animals.

Physiological changes in the brain, for instance, a dysregulation of the endocannabinoid receptors, may explain AD-related behavioral deficits. In the present study, we described that the increase in CB2 and GPR55 receptors in the hippocampus of HZ 5xFAD mice is involved in the anxiogenic response in the NOR test. Moreover, the reduction in CB1 together with the increase in GPR55 receptor, both aggravated by the transgenic load, was associated with hyperactivity when HZ 5xFAD mice were exposed to two identical objects in the acquisition trial of the NOR test. Furthermore, memory impairment was associated with this unbalanced expression of CB1, CB2, and GPR55 receptors found in HTZ and HZ 5xFAD mice. In the literature, recent evidence suggests that the stimulation of the CB2 receptor modulates neuronal function, as well as emotional behavior and memory formation [23]. Additionally, the upregulation of CB2 observed only in homozygosity is in line with previous publications that use the 5xFAD mice model [7], and it was corroborated in AD patients [21]. Regarding CB1, previous reports described that these receptors are highly expressed throughout the brain such as the hippocampus, with a notable presence on multiple neuronal populations (for a review, see [12]). In this context, data available suggest that CB1 receptors play an integral role in learning and memory showing a special relevance on anxiety-like behavior [14,55] and long-term (acquisition, consolidation, and retrieval) memory-relative responses [23,56,57,58,59,60]. Therefore, pharmacological modulation of endocannabinoid receptors may be a potential target for the treatment of AD, and attention has been paid to CB2 receptor-specific agonists because of their lack of psychotropic properties compared to among the CB1 receptor agonists [61]. In this sense, several studies support that the pharmacological treatment with the specific CB2 agonist JWH-133 ameliorates cognitive function and long-term recognition memory decline in AD-model animals, such as AβPP/PS1 mice [27,30,62], and APP 2576 mice [29] at 6 and 11 months of age, respectively. Although these results are promising, detailed studies are still needed. 

Regarding the noncannabinoid GPR55 receptor, we have to highlight that this receptor is primarily an LPI receptor, and we did not analyze the biochemical pathway for the synthesis or degradation of this bioactive lipid tightly related to endogenous cannabinoids. However, since GPR55 activity can be modulated by both endogenous and synthetic cannabinoids, we included it in the analysis. The GPR55 receptor has been previously described as being involved in spatial learning [33] and memory dysfunction [63], and GPR55 knockout mice showed impaired movement coordination [64]. Few studies investigated its pharmacological modulation to improve cognition [33], synapsis plasticity [65], and neural stem-cell proliferation [66], which may present a new target for the treatment of neurodegenerative diseases such as AD. An interesting hypothesis for the importance of GPR55 is not only its location on immune cells but specifically its intracellular location in the lysosomal compartment. Intracellular delivery of LPI can activate Ca^2+^ release from these organelles through a GPR55-dependent mechanism. This calcium release might disrupt the pH homeostasis of the lysosomal compartment because of the well-known mutual interaction of Ca^2+^ and H^+^ in these organelles [67,68]. Since lysosomal de-acidification is a relevant process associated with AD [69], the GPR55 modulation of AD progression hypothesis has to be considered in futures studies. It is currently unknown whether GPR55 modulates Aβ production and trafficking because there are no studies with selective GPR55 ligands (most of the studies were done using nonselective ligands such as the GPR18/GPR55 ligand abnormal cannabidiol). In any case, the contribution of GPR55 to the neuropathology of AD is supported by additional reports suggesting that LPI deposits are enriched in the outer layer of the amyloid plaque, facilitating the interaction with microglia/macrophages and probably modulating neuroinflammation [70]. In this sense, it is important to note that the association of LPI/GPR55 with degenerative diseases displays growing relevance. As an example, recent studies identified a crucial role for LPI signaling in metabolic disorders leading to nonalcoholic steatohepatitis, a degenerative disorder of the liver that leads to steatosis and fibrosis in which both GPR55 and LPI are upregulated [71]. Our findings set in place its expression in the hippocampus as a marker of severity, helping to clearly differentiate HTZ from HZ animals. Its association with the emotional and memory performance of HZ animals is very interesting, as well as its inverse correlation with neuroinflammatory markers from microglia, suggesting a potential anti-inflammatory role. Taken together, these data expose the importance of stimulating or inhibiting ECB receptors to ameliorate behavioral changes related to AD such as anxiety, memory deficit, or coordination of locomotion. 

Endocannabinoids, e.g., AEA or 2-AG, are synthesized and released by neurons acting as neurotransmitters. Nonetheless, ECB differentiates from other neurotransmitters because (i) retrograde signaling is their principal mode of acting as messengers [72], and (ii) they do not accumulate within synaptic vesicles [73]. Interestingly, neuronal damage boosts the production of ECB, providing a mechanism of protection against toxicity [74]. Once released upon demand, AEA and 2-AG activate the presynaptic cannabinoid receptors, and then they are rapidly inactivated by the action of specific degradation enzymes (FAAH for AEA, and MAGL for 2-AG) [75]. When analyzing production/degradation enzymes, we have seen that, overall, the activity of the enzymes involved in biosynthesis and degradation of ECBs is altered; there is an increase in production rather than in degradation in the HZ group. These findings may be related to the neuroinflammation observed in aged 5xFAD animals, which is clearly associated with the genetic load. The balance between production and degradation, measured as the DAGL/MAGL (for 2-AG) and NAPE-PLD/FAAH (for anandamide and other acylethanolamines) ratios favors ECB production. The activation of DAGLα and the reduction in MAGL activity may be increasing the 2-AG levels, thus leading to the desensitization of CB1 receptors [76], which could explain the reduced CB1 found in our samples. Several related works aligned with this hypothesis have been published supporting this idea, such as the work of Mulder et al. (2011) who determined the alteration of 2-AG signaling during late stages in the transgenic APdE9 mice [77]. Furthermore, Altamura et al. (2015) found that AD patients present high plasma 2-AG levels compared to controls [78]. Here, we propose an alteration of 2-AG signaling in the transgenic 5xFAD mice due to the combination of impaired MAGL degradation and increased DAGL levels, which is also a marker of severity, since it is only observed in HZ animals. Nonetheless, it will be necessary to measure the release of 2-AG in the hippocampus of these animals to confirm whether this marker is functionally relevant. The overall significance of this overproduction of endogenous cannabinoids could probably reflect an anti-inflammatory response to fight the damage associated with β-amyloid deposition. 

Currently, neuroinflammation is one of the hallmarks of AD pathology acting as a vicious cycle [79]. Cytotoxicity stimulatory factors such as Aβ deposition lead to microglial activation, releasing inflammatory and neurotoxic factors. This, in turn, causes progressive neuronal loss and degeneration, secreting neurotoxic factors and entering again into the AD vicious cycle. After evaluating inflammatory markers, we have seen that there is a substantial neuroinflammatory response in the hippocampus of these animals. Both Western blot results and immunohistochemistry staining show the accumulation of GFAP- and Iba1-positive cells within the hippocampus, being higher in the HZ than in the HTZ group. As expected, a higher transgenetic load leads to a more robust inflammatory response. GFAP is the main astrocytic intermediate filament considered to be a highly specific marker for glia [80], and Iba1 is a microglia/macrophage-specific calcium-binding protein [81]. Both have been previously studied in AD mouse models, suggesting that AD pathogenesis is not restrained to neurons but actively interacts with micro- and astroglia, triggering an innate immune response [82]. Another inflammatory marker analyzed was iNOS, which was highly expressed in the hippocampal cells of HZ mice. Interestingly, this oxidative stress marker has also been found in AD human samples, being involved in the pathogenesis of neuronal degeneration of the disease [83]. Here, we report the correlation between the expression of cannabinoid receptors and the substantial increase in neuroinflammation. Results show that a lower number of CB1 and an increasing number of CB2 receptors, both aggravated by the transgenic load, are related to the neuroinflammatory response. Overall, CB1 and GPR55 negatively correlate with the inflammatory markers, and CB2 positively correlates with them. This means that an increased genetic load in the animal model also increases the levels of CB2 receptor expression, which in turn is related to high levels of neuroinflammation since these receptors are mainly located in activated microglia. From these analyses, we can propose ECB receptors as biomarkers for inflammatory responses associated with AD. The potential utility of cannabinoid receptor ligands to modulate these inflammatory responses places them as new targets for developing therapies capable of acting as disease modifiers that retard the progression of the disease. A proof of concept of this neuroinflammation modulation is granted in this 5xFAD mouse model.

On the other hand, a negative correlation between CB1 expression and amyloid plaques (Aβtotal, Aβ40, and Aβ42) in the hippocampus of 5xFAD mice was reported. These results are consistent with other studies affirming that CB1 activity is higher at earlier AD stages and decrease at advanced stages [84]. Nevertheless, the increase in CB2 receptor in our HZ 5xFAD mice positively correlated with Aβ accumulation and senile plaque score. This is a response to excessive neuroinflammation induced by Aβ deposition, indicating an enhanced glial reactivity as confirmed by expanding previously published literature [7,19,21,85]. It is important to note that pharmacological evidence suggests that the CB2 receptor can modulate Aβ and hyperactivity tau levels [61], although there are conflicting results. The chronic treatment of a specific agonist of CB2, JWH-133, failed to reduce Aβ in the hippocampus and cortex of 5xFAD mice [27], while prolonged oral administration of this agonist reduced significantly cortical β-amyloid in Tg App 2576 mice [29]. These differences could be due to the animal model used and the severity of the disease. Moreover, Vázquez et al. (2015) found that, by blocking CB1, inflammation worsened in 5xFAD mice but no quantification of Aβ was specified [36]. Therefore, more experiments are needed to clarify the functionality of these receptors on the Aβ-clearance. Remarkably, the increased expression of the GPR55 receptor, depending on the complete transgene load, cannot be explained by Aβ accumulation, and it is independent of neuroinflammation. 

Finally, an important limitation of the present study is that the 5xFAD model is an amyloid deposition model that lacks a very relevant pathophysiological mechanism in AD: the deposition of hyperphosphorylated tau protein, responsible for neurofibrillary tangles. Further studies must be addressed in preclinical models of tau deposition to clarify the role of the endogenous cannabinoid system in AD, where both Aβ accumulation and tau deposition contribute to the loss of neurons and, subsequently, to the devastating dementia characterizing the disease.

## 5. Conclusions

Overall, the results of the present study suggest that the endocannabinoid system is involved in modulating memory impairment and anxiety-like behavior, as well as pathological changes in the 5xFAD mouse model such as inflammation. This modulatory response is related to the imbalance of the expression pattern of CB1, CB2, and the GPR55 receptors in the hippocampus. This imbalance is clearly aggravated by the transgenic load, making these receptors candidate markers for the severity of the disease. Here, we described for the first time the alteration of the ECB system in 5xFAD mice, taking the heterozygosity and homozygosity into special consideration, shedding light on the unknown neurobiological changes of the AD. However, more studies are needed to elucidate the role(s) of the ECB system and provide a new therapeutic target for the treatment of neurodegenerative diseases such as AD. In future work, we plan to investigate the release and dynamics of ECB in the brain of these animals through the development of the disease and to include a more complete protein–protein interaction pathway according to the literature. Understanding the evolution of the ECB system in these animals at an early stage will help to understand when and how to use new cannabinoid-based drugs to modify the course of the disease. Finally, using models where tau deposition is also present will help to better clarify the efficacy of this newly proposed ECB-based therapeutic approach to Alzheimer’s disease.

## Figures and Tables

**Table 1:** Pearson’s correlation between endocannabinoid receptors (CB1, CB2, and GPR55) and behavioral parameters related to memory measured by novel object recognition test in 5xFAD transgenic mice.

Pearson’s Correlation	CB1	CB2	GPR55
Time spent in the center: *habituation trial*	0.589	−0.523	−0.750
	0.72550	<0.0001 ***	<0.0001 ***
Locomotion (distance moved): *acquisition trial*	−0.479	0.445	0.680
	0.00210 **	0.45070	0.0249 *
Percentage novelty preference: *retention trial*	0.634	−0.483	−0.525
	0.03950 *	0.00340 **	0.0126 *

Pearson’s correlation coefficients (*r*) are shown above, and *p*-values are shown below. (*) *p* < 0.05; (**) *p* < 0.01; (***) *p* < 0.001.

**Table 2:** Pearson’s correlation between endocannabinoid receptors (CB1, CB2, and GPR55) and neuroinflammation-related proteins measured by Western blot analysis in 5xFAD transgenic mice hippocampus.

Pearson’s Correlation	CB1	CB2	GPR55
Iba1	−0.513	0.470	−0.704
	0.003 **	0.9685	0.0089 **
GFAP	−0.722	0.583	−0.656
	<0.0001 ***	0.0062 **	0.1453
COX2	−0.717	0.576	−0.696
	0.0006 ***	0.0446 *	0.0029 **
iNOS	−0.724	0.583	−0.729
	<0.0001 ***	0.716	0.0065 **

Pearson’s correlation coefficients (*r*) are shown above, and *p*-values are shown below. (*) *p* < 0.05; (**) *p* < 0.01; (***) *p* < 0.001.

**Table 3:** Pearson’s correlation between endocannabinoids receptors (CB1, CB2, and GPR55) and the β-amyloid burden (Aβ40, Aβ42, and Aβtotal) in the hippocampus of the 5xFAD transgenic mice.

Pearson’s Correlation	CB1	CB2	GPR55
Aβ40	−0.700	0.553	0.626
	<0.0001 ***	<0.0001 ***	0.4077
Aβ42	−0.723	0.587	0.712
	<0.0001 ***	<0.0001 ***	0.4806
Aβtotal	−0.717	0.575	0.681
	<0.0001 ***	<0.0001 ***	0.4652

Pearson’s correlation coefficients (*r*) are shown above, and *p*-values are shown below. (***) *p* < 0.001.

## Supplementary Materials

The following are available online at https://www.mdpi.com/2079-7737/9/11/377/s1: Table S1: Primary antibodies used for Western blotting and IHC, Figure S1: GPR55 immunofluorescence on 5xFAD mice hippocampus with its negative control shown below, Figure S2: One whole membrane was used for DAGLα, α-adaptin, CB1, FAAH, CB2, and MAGL immunoblotting on 5xFAD mice hippocampus (unedited blots, Figure 3 and Figure 4), Figure S3: One whole membrane was used for COX-2, α-adaptin, GFAP, and GPR55 immunoblotting on 5xFAD mice hippocampus (unedited blots, Figure 3 and Figure 5), Figure S4: One whole membrane was used for iNOS, α-adaptin, and NAPE-PLD immunoblotting on 5xFAD mice hippocampus (unedited blots, Figure 4 and Figure 5), Figure S5: One whole membrane was used for Iba1 and α-adaptin immunoblotting on 5xFAD mice hippocampus (unedited blots, Figure 5).

## Abbreviations

Aβ	β-Amyloid
Iba1	Ionized calcium-binding adaptor molecule 1
MAGL	Monoacylglycerol lipase
GPR55	G-protein-coupled receptor 55
CB2	Cannabinoid receptor type 2
NAPE-PLD	*N*-Acyl phosphatidylethanolamine-specific phospholipase D
GFAP	Glial fibrillary acidic Protein
CB1	Cannabinoid receptor type 1
FAAH	Fatty acid amide hydrolase
DAGLβ	Diacylglycerol lipase β
COX-2	Cyclooxygenase 2
DAGLα	Diacylglycerol lipase α
iNOS	Inducible nitric oxide synthase
LPI	Lysophosphatidylinositol
