# Supplementary material for: Imbalance of Endocannabinoid/Lysophosphatidylinositol Receptors Marks the Severity of Alzheimer’s Disease in a Preclinical Model: A Therapeutic Opportunity
Source: Biology (Basel). 2020 Nov 5;9(11):377. doi: 10.3390/biology9110377 (PMC7694492; doi:10.3390/biology9110377)

Supplementary material

Table S1. Primary antibodies used for Western blotting and IHC

Primary antibody	Manufacturing details	Dilution
**Iba1**	Wako Chemicals(#019-19741)	1/500
**MAGL**	Cayman Chemical (#100035)	1/500
**GPR55**	Abcam (#ab41515-100)	1/500
**CB2**	Abcam (#ab3561)	1/200
**NAPE-PLD**	Abcam (#ab95397)	1/1000
**GFAP [G3893]**	Sigma-Aldrich (#083M4785)	1/500
**CB1**	Abcam (#ab23703)	1/100
**FAAH**	Cayman Chemical (#101600)	1/100
**DAGLβ**	bioNova Científica (#orb182976)	1/100
**COX-2 [D5H5]**	Cell Signaling Technology (#12282)	1/200
**α-Adaptin [ERP7572]**	Abcam (ab151720)	1/2000
**DAGLα**	bioNova Científica (#orb156533)	1/100
**iNOS**	Thermo Fisher Scientific (#PA1-036)	1/200
**beta Amyloid (1-42)**	Thermo Fisher Scientific (#44-344)	1/200
**beta Amyloid (1-40)**	Thermo Fisher Scientific (#44-136)	1/200
**beta Amyloid**	Abcam (#ab2539)	1/200

Abbreviations: Iba1, Ionized calcium binding adaptor molecule 1; MAGL: Monoacylglycerol lipase; GPR55: G protein-coupled receptor 55; CB2: Cannabinoid receptor type 2; NAPE-PLD: N-acyl phosphatidylethanolamine-specific phospholipase D; GFAP, glial fibrillary acidic Protein; CB1: Cannabinoid receptor type 1; FAAH: Fatty acid amide hydrolase; DAGLβ: Diacylglycerol Lipase β; COX-2, Cyclooxygenase 2; DAGLα: Diacylglycerol Lipase α; iNOS: Inducible nitric oxide synthase.

Figure S1: GPR55 immunofluorescence on 5xFAD mice hippocampus with its negative control shown below.

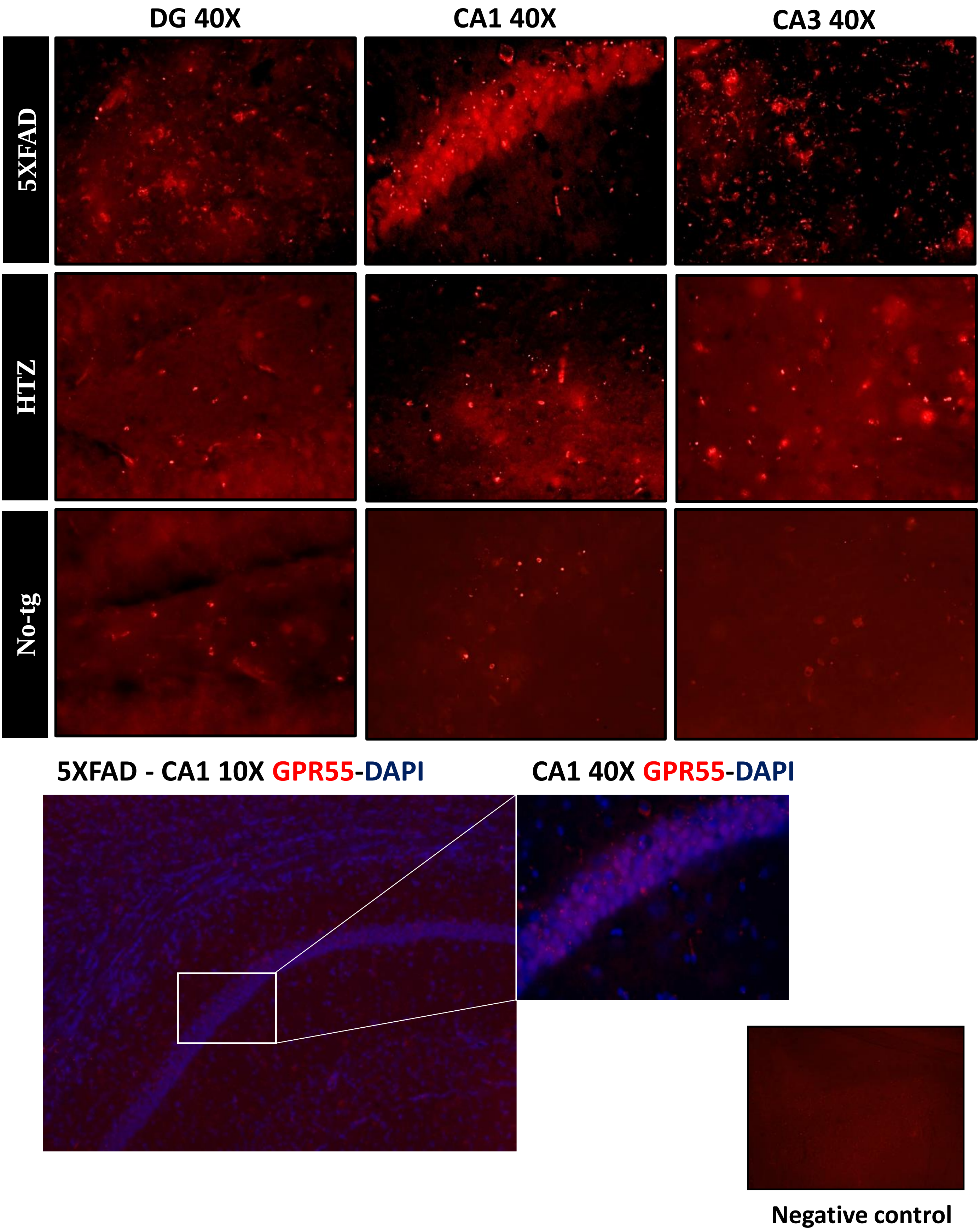

Figure S2 (unedited blots): One whole membrane was used for DAGLα, α-Adaptin, CB1, FAAH, CB2 and MAGL immunoblottings on 5xFAD mice hippocampus (Figure 3&4)

Whole membranes from Gel 1. Individual membranes are shown below. **CB1** and **CB2** are represented in Figure 3a&b; **DAGLα** is represented in Figure 4a&d; **FAAH** and **MAGL** are represented in Figure 4b&d. All bands were quantified for histograms charts and statistical analysis. Proteins were normalized with α-Adaptin.

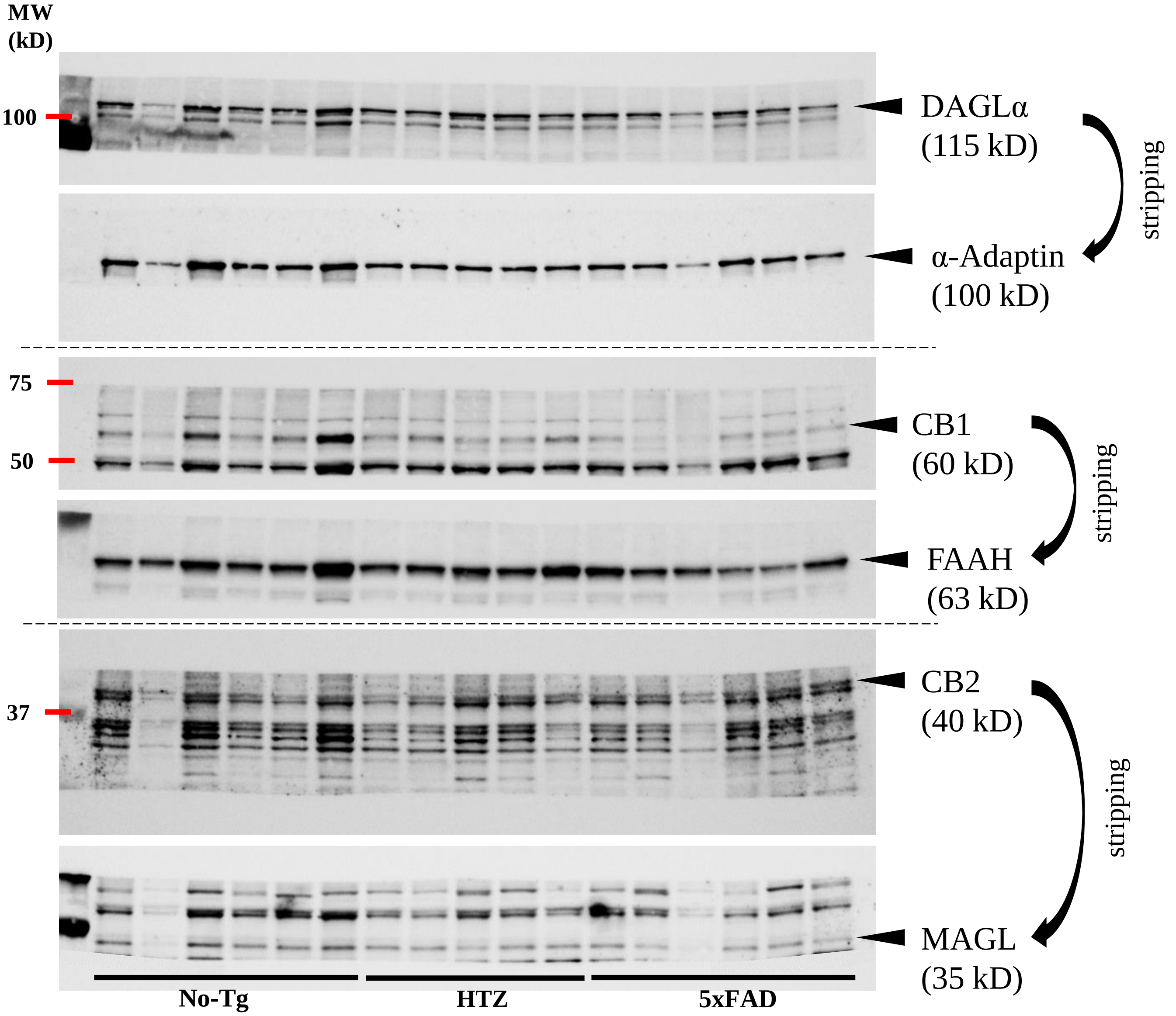

Figure S3 (unedited blots) : One whole membrane was used for COX-2, α-Adaptin, GFAP and GPR55 immunoblottings on 5xFAD mice hippocampus (Figure 3&5)

Whole membranes from Gel 2. Red Ponceau Staining and individual membranes are shown below. **GPR55** is represented in Figure 3a&b; **GFAP** is represented in Figure 5a&b; **COX-2** is represented in Figure 5f&g. All bands were quantified for histograms charts and statistical analysis. Proteins were normalized with α-Adaptin.

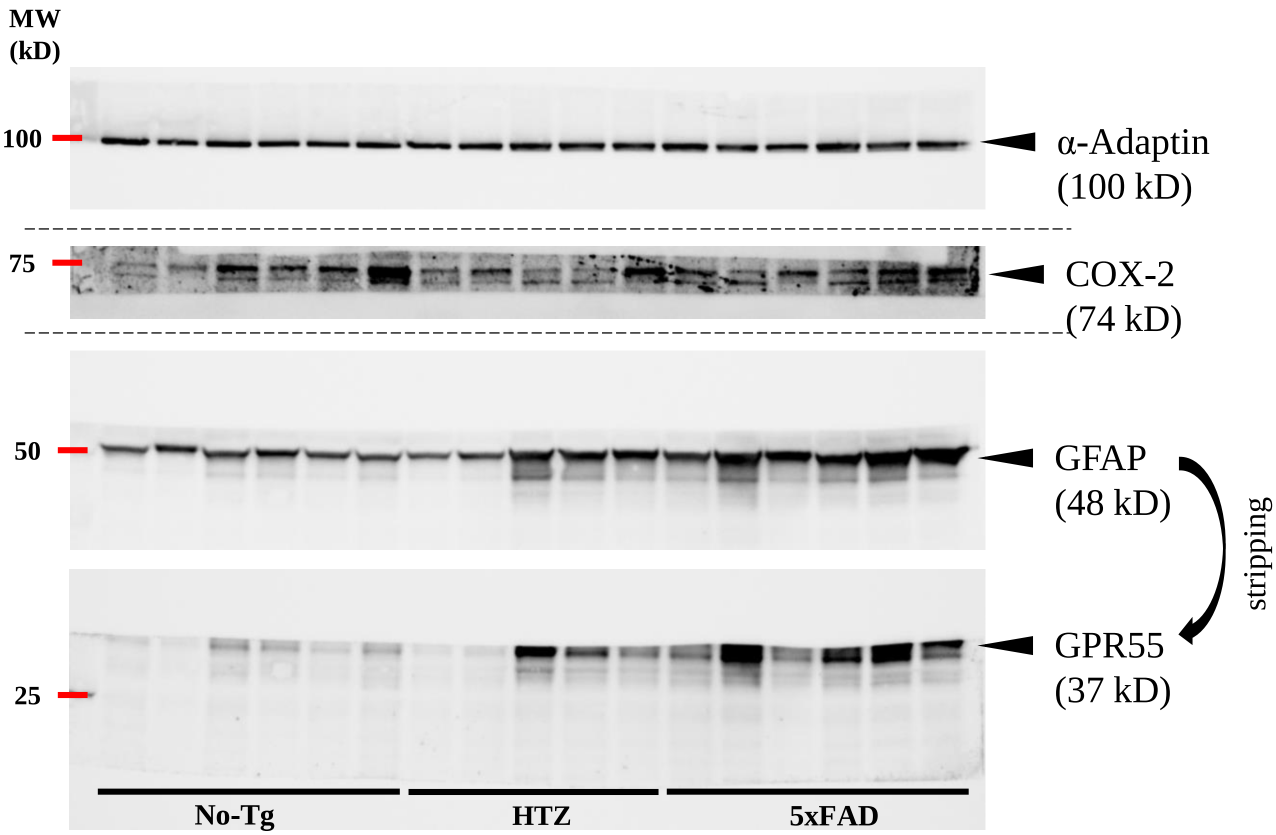

Figure S4 (unedited blots) : One whole membrane was used for iNOS, α-Adaptin, and NAPE-PLD immunoblottings on 5xFAD mice hippocampus (Figure 4&5)

Whole membranes from Gel 3. Red Ponceau Staining and individual membranes are shown below. **NAPE-PLD** is represented in Figure 4a&d; **iNOS is** represented in Figure 5f&g. All bands were quantified for histograms charts and statistical analysis. Proteins were normalized with α-Adaptin.

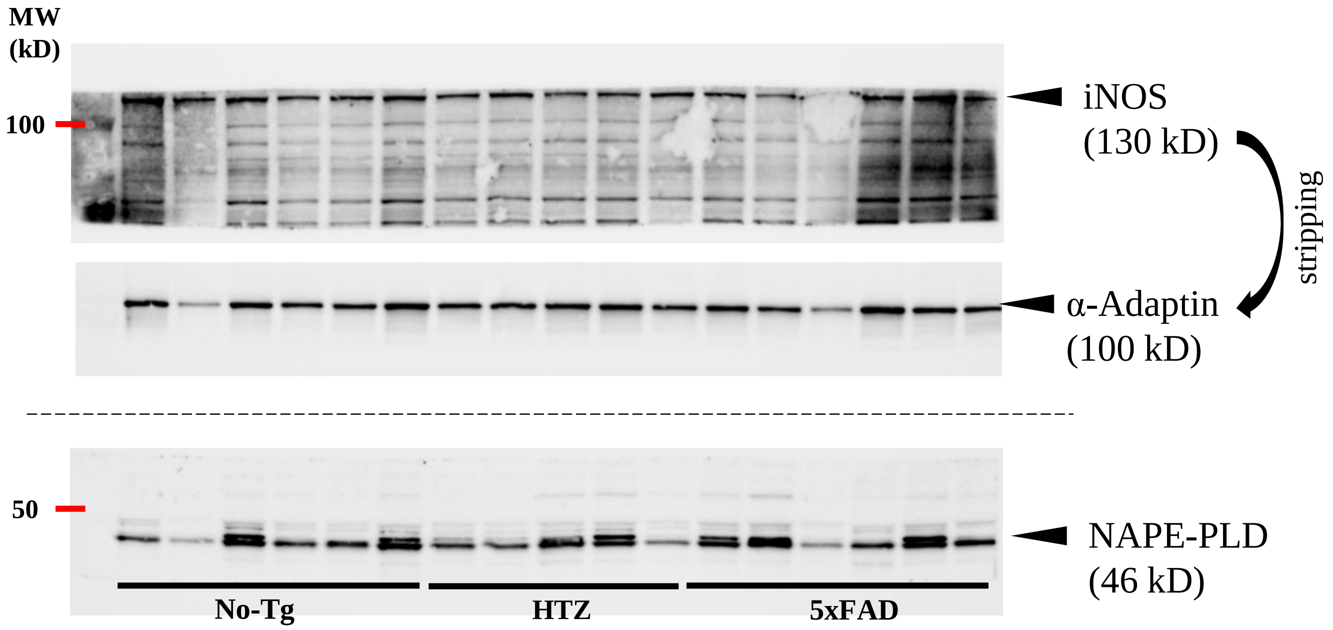

Figure S5 (unedited blots) : One whole membrane was used for Iba1 and α-Adaptin immunoblotting on 5xFAD mice hippocampus (Figure 5)

Whole membranes from Gel 4. Red Ponceau Staining and individual membranes are shown below. **Iba1 is** represented in Figure 5a&b. All bands were quantified for histograms charts and statistical analysis. Proteins were normalized with α-Adaptin.

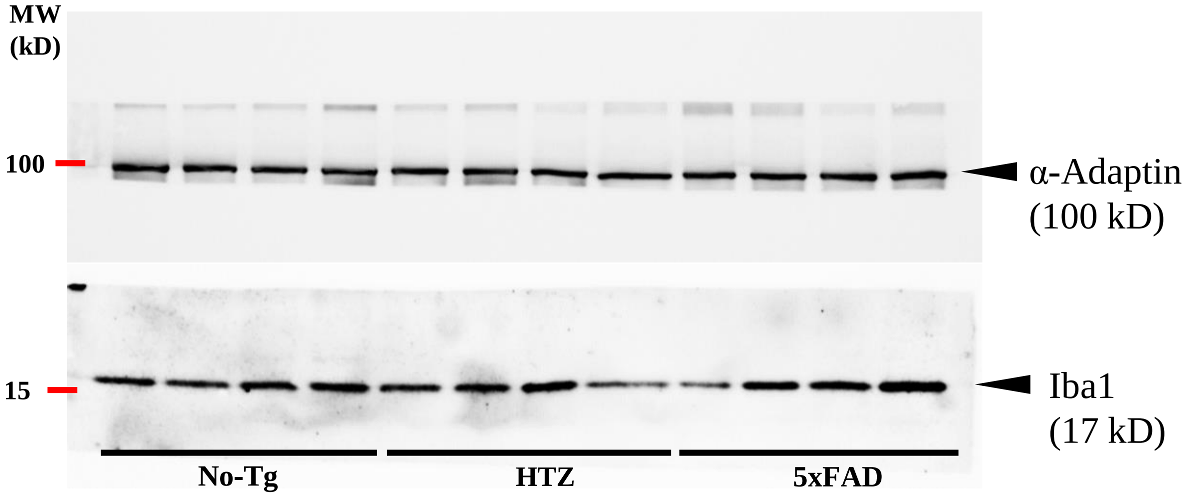

Supplement: Supplementary file 1 [file biology-09-00377-s001.pdf]
